# Radiotherapy Effects on Anti-Tumor Immunity: Implications for Cancer Treatment

**DOI:** 10.3389/fonc.2013.00128

**Published:** 2013-05-22

**Authors:** Sandra Demaria, Silvia C. Formenti

**Affiliations:** ^1^Department of Pathology, New York University School of Medicine, New York University Langone Medical CenterNew York, NY, USA; ^2^Department of Radiation Oncology, New York University School of Medicine, New York University Langone Medical CenterNew York, NY, USA

Ionizing radiation (IR) is a powerful therapeutic modality for cancer, commonly used for its capacity to kill cancer cells. In this Frontiers Research Topic Book radiotherapy effects are re-visited, from the point of view of the host's immune system (IS). An introductory article from Golden et al. (2012) examines the consequences of the many types of radiation-induced tumor cell death and how these coalesce to generate the key signals that define an immunogenic cell death (ICD). Cancer cells dying by ICD deliver a cascade of signals to the IS that culminates in the generation of anti-tumor T cells by providing a source of antigen for cross-presentation coupled with maturation signals to dendritic cells (DC). The ability of IR to induce an ICD is exploited by novel cancer therapies that have, for instance, shown the benefit of intra-tumoral injection of DC post-radiotherapy in preclinical models. Finkelstein and Fishman (2012) discuss this approach and the emergence of encouraging results from clinical pilot studies.

Burnette et al. (2012) provide an overview of the immunological environment existing in tumor-bearing hosts, emphasizing the challenge of overcoming tolerance and immunosuppression to achieve tumor rejection. To overcome this barrier, combinations of IR with specific immunotherapies have been tested by several labs and shown to be effective at eliciting robust anti-tumor immunity. One such strategy, discussed by Mason and Hunter (2012), is the combination of IR with intra-tumoral synthetic oligodeoxynucleotides such as CpG. The preclinical success of this combination was translated to the clinic where it has demonstrated to induce rejection of the irradiated tumor as well as tumors outside the radiation field (abscopal effect). Another strategy, which is in earlier stage of investigation but holds great potential, is nanovectorized radiotherapy discussed by Vanpouille-Box and Hindré (2012). Delivery of radionuclides using nanoparticles has the advantage of providing targeting specificity to the tumor as well as exploiting the intrinsic immunostimulatory properties of nanoparticles.

Importantly, IR effects exceed the classical cytocidal properties by also causing phenotypic changes in the fraction of surviving cells, markedly enhancing their susceptibility to T cell-mediated elimination. Kwilas et al. (2012) define these effects of IR as “immunogenic modulation” and illustrate the examples of IR-induced Major Histocompatibility Complex antigens and death receptors, which improve tumor rejection by T cells adoptively transferred or activated by vaccination.

However, not all IR-induced modifications of the tumor and its microenvironment favor immune rejection. Chiang et al. (2012) provide novel evidence for accumulation of pro-tumorigenic M2 macrophages in areas of hypoxia present in irradiated tumors. Schaue et al. (2012) discuss the increase of regulatory T cells post-radiotherapy, potentially hindering the development of effective anti-tumor T cell responses. Intriguingly, the dose and fractionation of radiotherapy may play a role in modulating the expansion of effector versus regulatory T cells. This aspect is critically addressed by Demaria and Formenti (2012). Since much of the available preclinical data come from experiments testing single IR doses, further exploration of fractionated regimens is warranted.

Overall, the book provides an overview of the available data and evolving concepts in support of a novel use of radiotherapy: that of an immune modulator and optimal partner for immunotherapy. While enthusiasm for the combination of IR and immunotherapy was enhanced by recent anecdotal reports in some cancer patients, much work remains to be done. Hopefully, the book will inspire more investigators to explore this new area, and encourage more discovery of the interaction of IR and immunity.
